# Subclinical hyperthyroidism and the risk of dementia: A meta‐analysis

**DOI:** 10.1002/brb3.70037

**Published:** 2024-09-18

**Authors:** Qiao Liu, Chaoyin Lu, Mengdie Chen, Ping Feng

**Affiliations:** ^1^ Department of Endocrinology Taizhou Central Hospital (Taizhou University Hospital) Taizhou China

**Keywords:** dementia, meta‐analysis, risk factor, subclinical hyperthyroidism, thyroid function

## Abstract

**Background:**

Accumulating evidence suggests that thyroid dysfunction may be related to the risk of dementia. However, previous studies evaluating the association between subclinical hyperthyroidism and the risk of dementia showed inconsistent results. This systematic review and meta‐analysis were performed to evaluate the relationship between subclinical hyperthyroidism and the incidence of dementia in the general population.

**Methods:**

Cohort studies relevant were retrieved by searching the electronic databases including PubMed, Web of Science, and Embase. A random‐effects model was used to combine the data by incorporating the influence of between‐study heterogeneity. Subgroup and meta‐regression analyses were performed to investigate the source of heterogeneity.

**Results:**

Nine cohort studies including 49,218 community‐derived participants were included. Among them, 3177 (6.5%) had subclinical hyperthyroidism at baseline. During a mean follow‐up of 10.2 years, 4044 participants developed dementia. The pooled results showed that compared to the participants with euthyroidism, those with subclinical hyperthyroidism had a higher incidence of dementia (risk ratio: 1.38, 95% confidence interval: 1.09 to 1.74, *p* = .006; *I* ^2^ = 47%). Subgroup analyses according to study design, age of the participants, methods for diagnosis of dementia, or analytic model did not significantly change the results. The univariate meta‐regression showed that the cutoff of thyroid‐stimulating hormone for defining subclinical hyperthyroidism negatively affected the association between subclinical hyperthyroidism and dementia (coefficient: –1.44, *p* = .009), which completely explained the heterogeneity (residual *I* ^2^ = 0%).

**Conclusion:**

Subjects with subclinical hyperthyroidism may have a higher risk of dementia compared to those with euthyroidism.

## Graphical abstract

A meta‐analysis incorporating nine cohort studies of 49218 community‐derived participants showed that subjects with subclinical hyperthyroidism may have a higher risk of dementia compared to those with euthyroidism. These results highlight the importance of the evaluation of thyroid dysfunction in the risk stratification of dementia in the general population, even for those with subclinical hyperthyroidism.

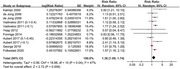

## 1 INTRODUCTION

Dementia is a debilitating condition characterized by cognitive impairment, which has been linked to heightened morbidity and mortality rates among the global population (Bransby et al., 2024; Cao et al., 2020; Smith & Ismail, 2021). Etiologically, dementia can be categorized into Alzheimer's disease (AD), vascular dementia (VaD), and other causes (Emrani et al., 2020; Raz et al., 2016). Identifying the risk factors associated with dementia is crucial for early prevention efforts (Campbell et al., 2013; Ranson et al., 2021). Subclinical hyperthyroidism is a milder form of hyperthyroidism, which has also been associated with multiple cardiovascular complications, especially when the thyroid stimulating hormone (TSH) level is below 0.1 mIU/L (Delitala, 2017; Smedegaard et al., 2020; Vidili et al., 2021). Accumulating evidence demonstrated that patients with hyperthyroidism may exhibit a higher risk of dementia compared to those with normal thyroid function (Joy Mathew et al., 2020; Khaleghzadeh‐Ahangar et al., 2022). Nevertheless, prior investigations examining the relationship between subclinical hyperthyroidism and the risk of dementia have yielded conflicting findings (van Vliet et al., 2021). Several preliminary studies have indicated a positive correlation between subclinical hyperthyroidism and an elevated incidence of dementia (Aubert et al., 2017; de Jong et al., 2009; Folkestad et al., 2020; Kalmijn et al., 2000; Vadiveloo et al., 2011), whereas other studies have failed to observe a similar association (de Jong et al., 2006; Formiga et al., 2014; George et al., 2019; Yeap et al., 2012). Although the underlying reasons for the inconsistent results are still not known, differences in study design, characteristics of the participants, cutoff of TSH for the diagnosis of subclinical hyperthyroidism, and definition of dementia outcome may affect the results. Here, we performed a systematic review and meta‐analysis to comprehensively evaluate the relationship between subclinical hyperthyroidism and dementia in general adult population and to explore the potential study characteristics on the results.

## 2 METHODS

The new edition of Preferred Reporting Items for Systematic reviews and Meta‐Analyses (PRISMA) statement (2020) (Page et al., 2021) were followed in this study. The Cochrane's Handbook (Higgins et al., 2021) for systematic review and meta‐analysis was referenced throughout the study.

### 2.1 Literature analysis

Three major electronic databases including PubMed, Web of Science, and Embase were used for literature search with a predefined combined search term including “hyperthyroidism,” “subclinical hyperthyroidism,” “thyroid function,” “thyroid diseases,” and “thyroid hormones” combined with “dementia,” “Alzheimer,” and “cognitive.” Only studies including human subjects were considered and no restriction was applied to the publication language. A second‐round check‐up for the references of the relevant articles was also conducted. The final search for the three databases was performed on January 5, 2024.

### 2.2 Inclusion and exclusion criteria

Inclusion criteria:

1. Cohort studies in full‐length articles;
2. Studies included the general adult participants;
3. Subclinical hyperthyroidism was considered as the exposure at baseline;
4. The incidence of all‐cause dementia, AD, or VaD were observed during follow‐up and compared between participants with subclinical hyperthyroidism and normal thyroid function;
5. The relative risk for the relationship between subclinical hyperthyroidism and dementia outcome was reported.

Subclinical hyperthyroidism could be diagnosed by either of the criteria that were consistent with those used in the original studies. Reviews, preclinical studies, retrospective or cross‐sectional studies, or studies without outcome of interest were excluded.

### 2.3 Data collection and quality assessment

Two independent authors conducted literature search and analysis, data collection, and study quality assessing separately. If discrepancies were encountered, the corresponding author joined the discussion for final judgment. Data of study information, participant demographic factors, cutoff of thyroid‐stimulating hormone (TSH) for the diagnosis of subclinical hyperthyroidism, follow‐up durations, and methods for validation of dementia outcomes were collected. Study quality assessment was achieved via the Newcastle–Ottawa Scale (Wells et al., 2010) with scoring regarding the criteria for participant selection, comparability of the groups, and the validity of the outcomes. The scale ranged between 1 and 9 stars, with larger number of stars presenting higher study quality.

### 2.4 Statistical strategy

The relative risk for dementia between participants with subclinical hyperthyroidism and normal thyroid function was presented with risk ratios (RRs) as well as their confidence intervals (CIs). Using the 95% CIs or *p* values, data of RRs and the standard errors (SEs) could be calculated, and a subsequent logarithmical transformation was conducted to maintain stabilized variance and normalized distribution (Higgins et al., 2021). The between study heterogeneity was estimated with the Cochrane's *Q* test and the *I* ^2^ statistic (Higgins & Thompson, 2002; Patsopoulos et al., 2008), with *I* ^2^ > 50% reflecting the significant heterogeneity. A random‐effect model was applied to combine the results by incorporating the influence of heterogeneity (Higgins et al., 2021). Sensitivity analysis by excluding one study at a time was used to evaluate the robustness of the finding (Higgins et al., 2021). Subgroup analyses were also performed to explore if participant or study feature may affect the results, such as study design, age of the participants, diagnostic methods for dementia, and analytic model (univariate or multivariate regression), if adequate datasets were available. In addition, a univariate meta‐regression was performed to evaluate the influence of TSH cutoff for the diagnosis of subclinical hyperthyroidism on the association between subclinical hyperthyroidism and the risk of dementia (Higgins et al., 2021). By construction of the funnel plots, the publication bias was estimated based on the visual judgment of the symmetry of the plots, supplemented with the Egger's regression asymmetry test (Egger et al., 1997). The RevMan (Version 5.1; Cochrane Collaboration, Oxford, UK) and Stata (version 12.0; Stata Corporation, College Station, TX) software packages were applied for these analyses.

## 3 RESULTS

### 3.1 Study identification and inclusion

**FIGURE 1:**
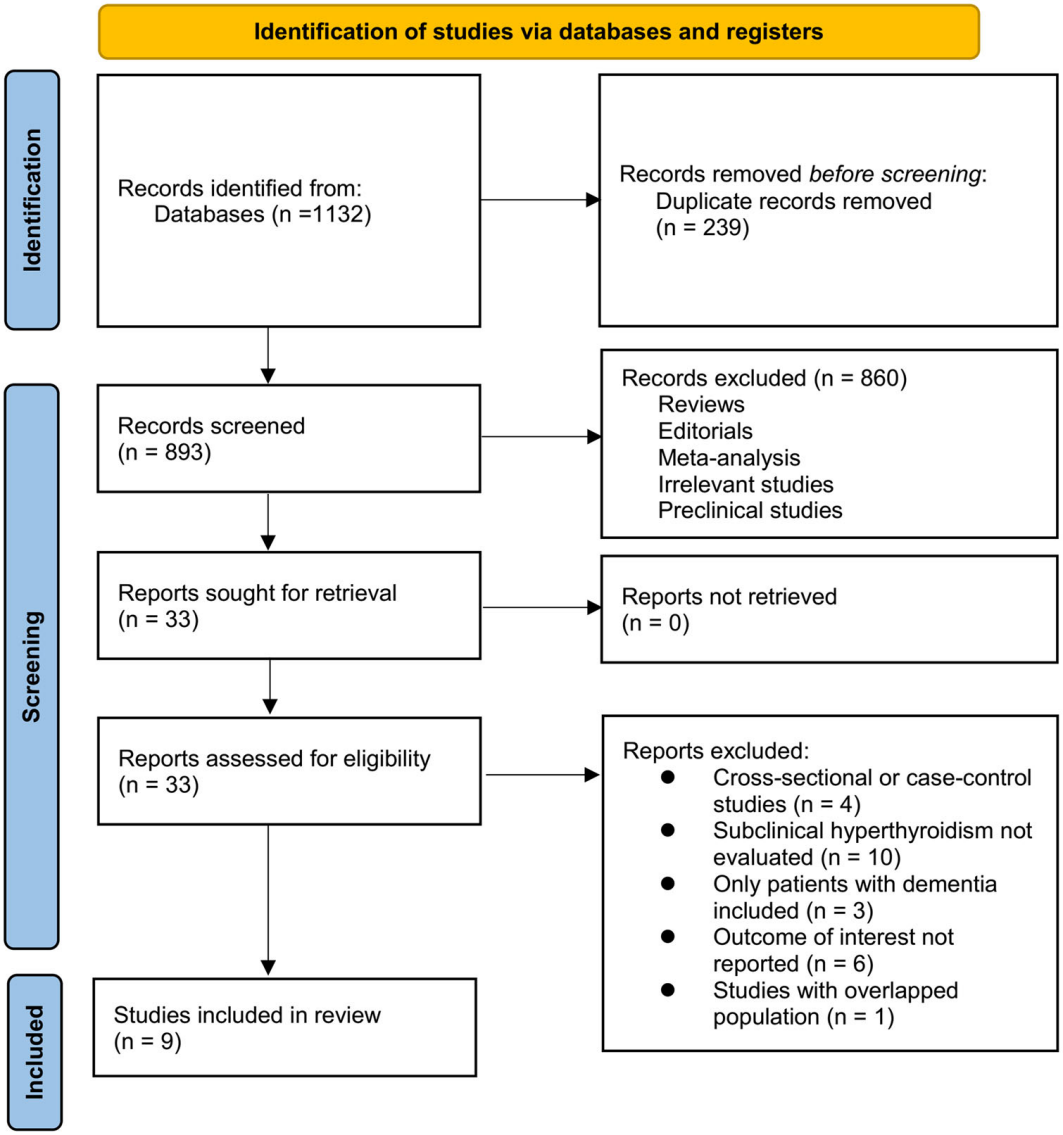
Process of literature search and study identification.

The flowchart for study identification and inclusion is shown in Figure 1. Briefly, we obtained a total of 1132 potentially relevant records after comprehensive searches of the three databases, and 239 of them were immediately excluded due to duplication. Subsequently, a screening via considering the titles and abstracts of the remaining records further led to the exclusion of 860 more studies, mostly because they were not related to the aim of the meta‐analysis. Accordingly, the full texts of the 33 remaining records were read by two independent authors, and 24 of them were further removed for various reasons, as listed in Figure 1. Finally, nine cohort studies remained suitable for the subsequent quantitative analyses (Aubert et al., 2017; de Jong et al., 2006, 2009; Folkestad et al., 2020; Formiga et al., 2014; George et al., 2019; Kalmijn et al., 2000; Vadiveloo et al., 2011; Yeap et al., 2012).

### 3.2 Overview of the studies’ characteristics

Table 1 presents the summarized characteristics of the included studies. Overall, nine cohort studies, comprising seven prospective cohort studies (Aubert et al., 2017; de Jong et al., 2006, 2009; Formiga et al., 2014; George et al., 2019; Kalmijn et al., 2000; Yeap et al., 2012) and two retrospective cohort studies (Folkestad et al., 2020; Vadiveloo et al., 2011), were included in the meta‐analysis. These studies were published between 2000 and 2020, and performed in the Netherlands, the United States, the United Kingdom, Australia, Spain, and Denmark. All of the studies included community‐derived population without dementia at baseline. The mean ages of the participants were 57−85 years, and the proportions of men were 22% to 100%. Different cutoffs of TSH were used for the diagnosis of subclinical hyperthyroidism, ranging from 0.1 to 0.56 mIU/L. Accordingly, 3177 (6.5%) participants had subclinical hyperthyroidism at baseline. During the mean follow‐up of 10.2 years, 4044 participants were diagnosed as dementia. The diagnosis of dementia was based on clinical evaluation in six studies (Aubert et al., 2017; de Jong et al., 2006, 2009; Formiga et al., 2014; George et al., 2019; Kalmijn et al., 2000) and via the International Classification of Diseases codes in three studies (Folkestad et al., 2020; Vadiveloo et al., 2011; Yeap et al., 2012). Two studies also reported the incidence of AD (de Jong et al., 2006; Kalmijn et al., 2000). Univariate analyses were used in three studies (de Jong et al., 2009; Formiga et al., 2014; Yeap et al., 2012) when the association between subclinical hyperthyroidism and the incidence of dementia was investigated. In the other six studies (Aubert et al., 2017; de Jong et al., 2006; Folkestad et al., 2020; George et al., 2019; Kalmijn et al., 2000; Vadiveloo et al., 2011), multivariate analyses were used and potential confounding factors were adjusted, such as age, sex, and comorbidities, to varying degrees. The NOS of the included studies were six to nine stars, suggesting overall moderate to good study quality (Table 2).

**TABLE 1:** Summary of study characteristics.

Author year	Country	Design	Population	Sample size	Mean age (years)	Men (%)	Mean FT4 at baseline (ng/dl)	TSH cutoff for Shyper (mIU/L)	No. of subjects with Shyper	Mean follow‐up duration (years)	Diagnosis of dementia	Number of patients with dementia	Variables adjusted
Kalmijn 2000	The Netherlands	PC	Community people aged 55 years or above	1843	68.8	38.1	NR	< 0.4	102	2.1	DSM‐III and NINCDS‐ADRDA	Overall: 25, AD: 18	Age and sex
de Jong 2006	The Netherlands	PC	Community people aged 60–69 years	1077	72.3	48.8	1.4	< 0.4	69	5.5	DSM‐III and NINCDS‐ADRDA	Overall: 63, AD: 46	Age and sex
de Jong 2009	The US	PC	Community men aged 71–93 years	1991	78.1	100	NR	< 0.4	24	4.7	DSM‐III	Overall: 106	None
Vadiveloo 2011	UK	RC	Community population	12115	66.3	22.6	NR	0.1−0.4 and < 0.1	2004	5.6	ICD codes	Overall: 204	Age, sex, and comorbidities
Yeap 2012	Australia	PC	Community men aged 70–89 years	3401	76.9	100	1.24	< 0.4	19	5.9	ICD codes	Overall: 145	None
Formiga 2014	Spain	PC	Community population aged 85 years at baseline	307	85	45.4	NR	< 0.45	5	3	Medical chart confirmed	Overall: 28	None
Aubert 2017	The US	PC	Community people aged 70–79 years	2558	75.1	48.2	NR	0.1−0.45 and < 0.1	80	9	Clinically diagnosed (significant decline in MMSE, dementia‐related prescription, or hospitalization)	Overall: 574	Age, sex, race, education level, cognition at baseline, and CVD risk factors
George 2019	The US	PC	Community population	12481	57	44.1	1.1	< 0.56	429	21.9	Clinically diagnosed (significant decline in MMSE, dementia‐related prescription, or hospitalization)	Overall: 2235	Age, sex, race‐center, APOE ε4, income and education, comorbidities and CVD risk factors
Folkestad 2020	Denmark	RC	Community population	13445	62.8	43.8	NR	< 0.3	445	7.3	ICD codes	Overall: 664	Age, sex, and CCI

TSH, thyroid‐stimulating hormone; Shyper, subclinical hyperthyroidism; PC, prospective cohort; RC, retrospective cohort; NINCDS‐ADRDA, National Institute of Neurologic and Communicative Disorders and Stroke/AD and Related Disorders Association; DSM‐III, Diagnostic and Statistical Manual of Mental Disorders, Revised Third Edition criteria; ICD, International Classification of Diseases; AD, Alzheimer's disease; CVD, cardiovascular diseases; CCI, Charlson Comorbidity Index; NR, not reported; FT4, free T4 (thyroxine).

**TABLE 2:** Study quality assessment using the Newcastle‐Ottawa Scale.

Study	Representativeness of the exposed cohort	Selection of the nonexposed cohort	Ascertainment of exposure	Outcome not present at baseline	Control for age and sex	Control for other confounding factors	Assessment of outcome	Enough long follow‐up duration	Adequacy of follow‐up of cohorts	Total
Kalmijn 2000	1	1	1	1	1	0	1	0	1	7
de Jong 2006	1	1	1	1	1	0	1	1	1	8
de Jong 2009	1	1	1	1	0	0	1	0	1	6
Vadiveloo 2011	0	1	1	1	1	1	0	1	1	7
Yeap 2012	1	1	1	1	0	0	0	1	1	6
Formiga 2014	1	1	1	1	0	0	1	0	1	6
Aubert 2017	1	1	1	1	1	1	1	1	1	9
George 2019	1	1	1	1	1	1	1	1	1	9
Folkestad 2020	0	1	1	1	1	1	0	1	1	7

### 3.3 Results of the meta‐analysis

**FIGURE 2:**
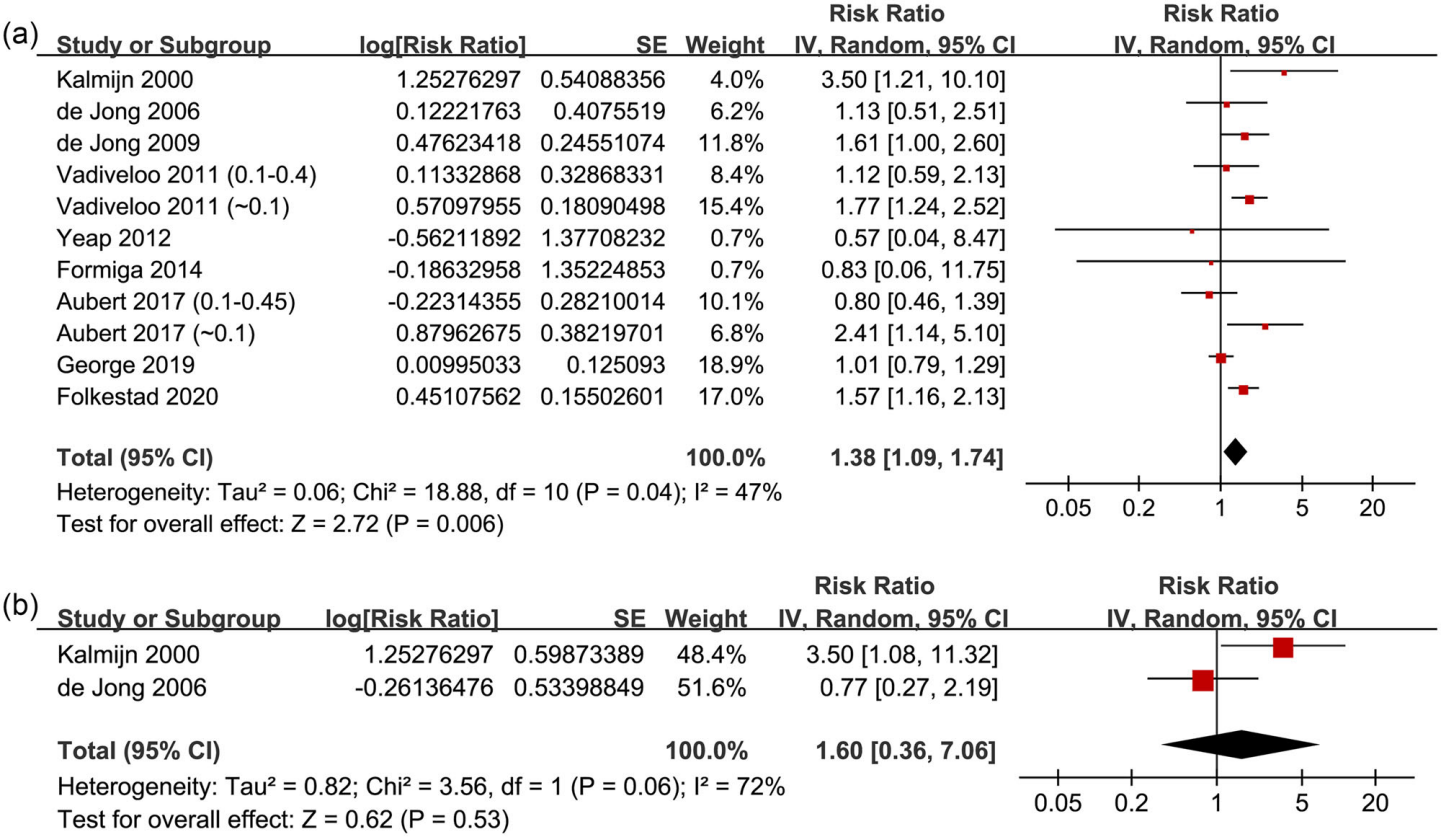
Forest plots for the meta‐analysis of the association between subclinical hyperthyroidism and dementia: (A) forest plots for the meta‐analysis of the outcome of overall dementia and (B) forest plots for the meta‐analysis of the outcome of AD.

**FIGURE 3:**
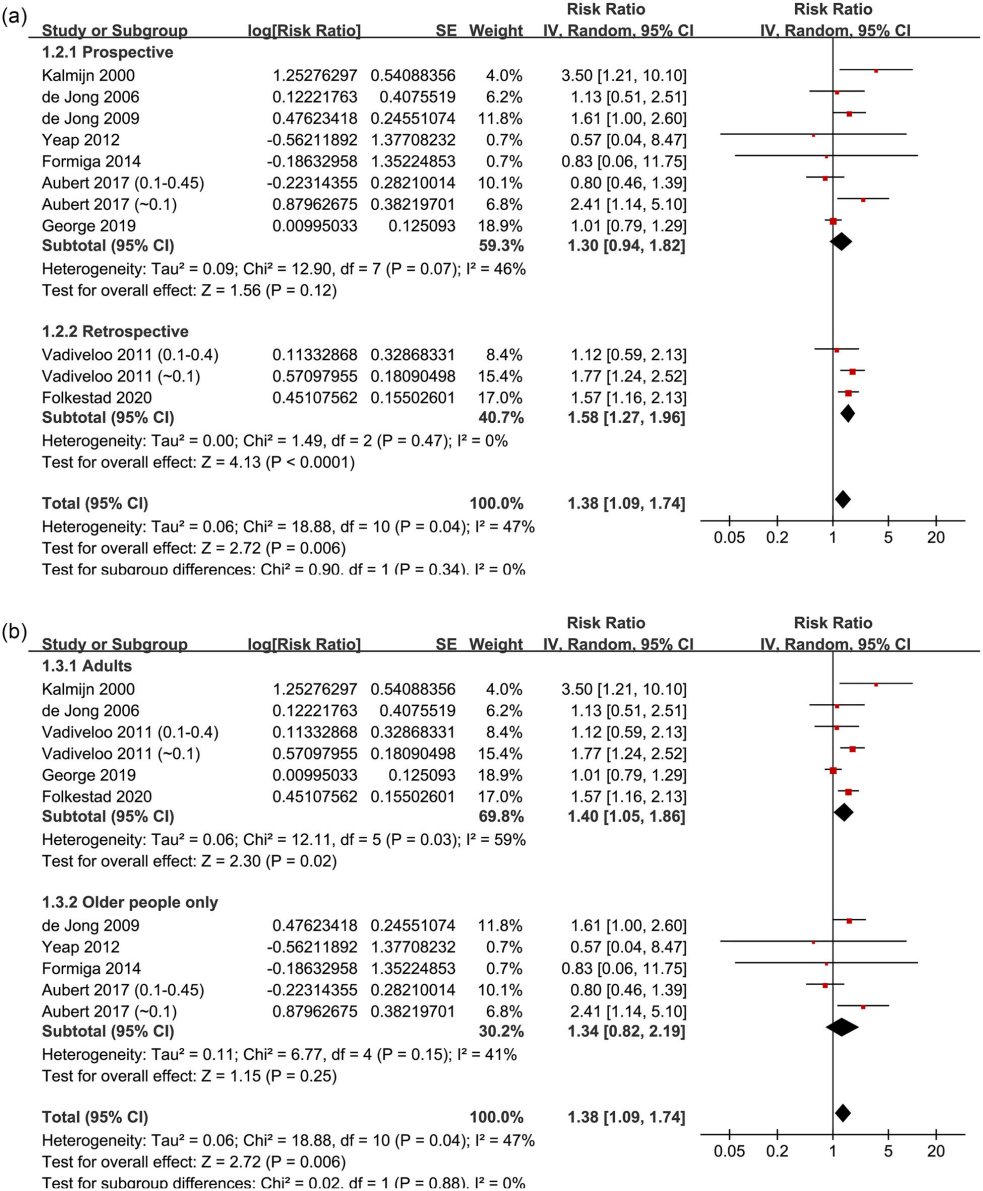
Forest plots for the subgroup analyses of the association between subclinical hyperthyroidism and the risk of overall dementia: (A) subgroup analysis according to study design and (B) subgroup analysis according to age group of the participants.

**FIGURE 4:**
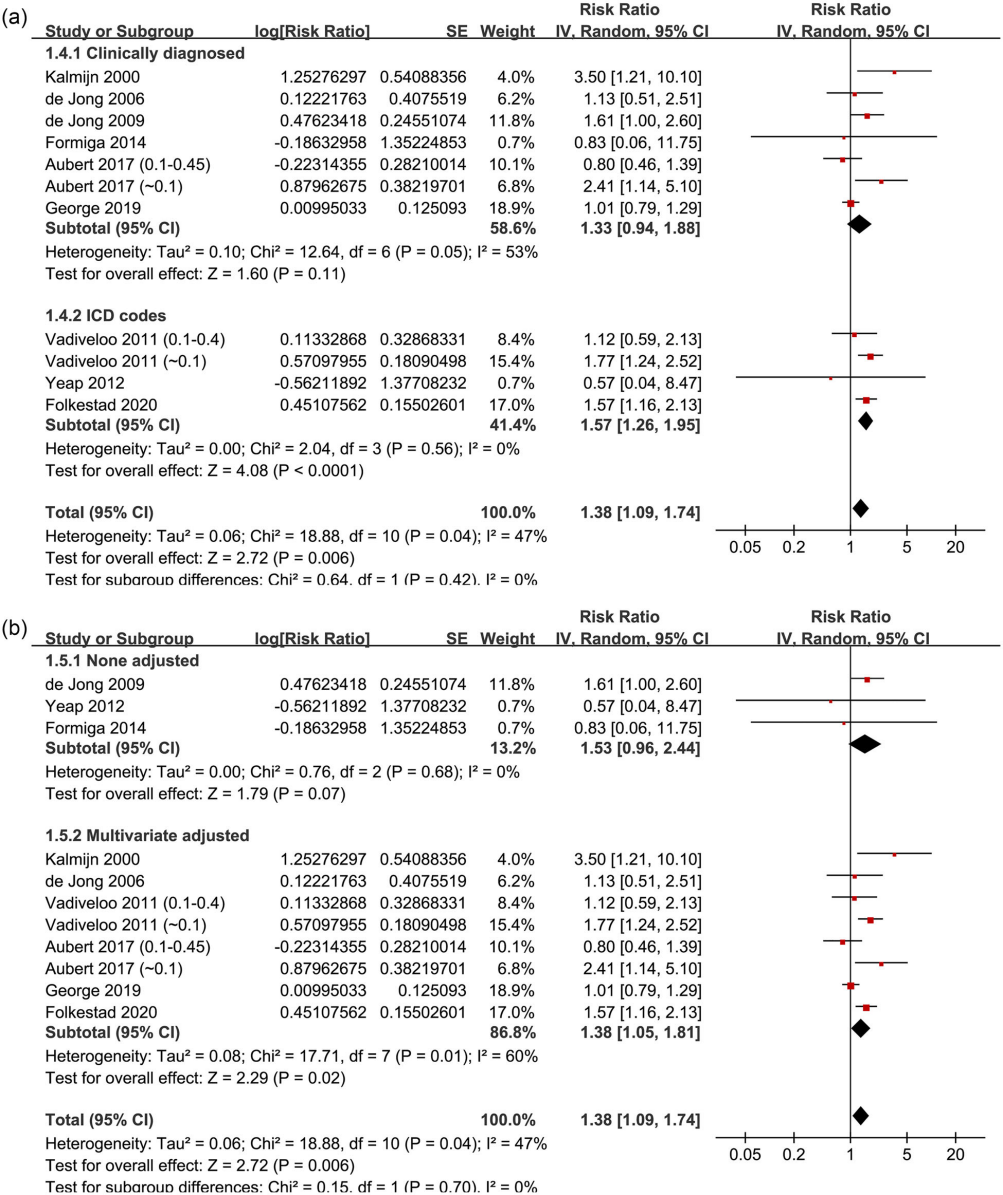
Forest plots for the subgroup analyses of the association between subclinical hyperthyroidism and the risk of overall dementia: (A) subgroup analysis according to the methods for the diagnosis of dementia and (B) subgroup analysis according to the regression model used for the analyses.

**FIGURE 5:**
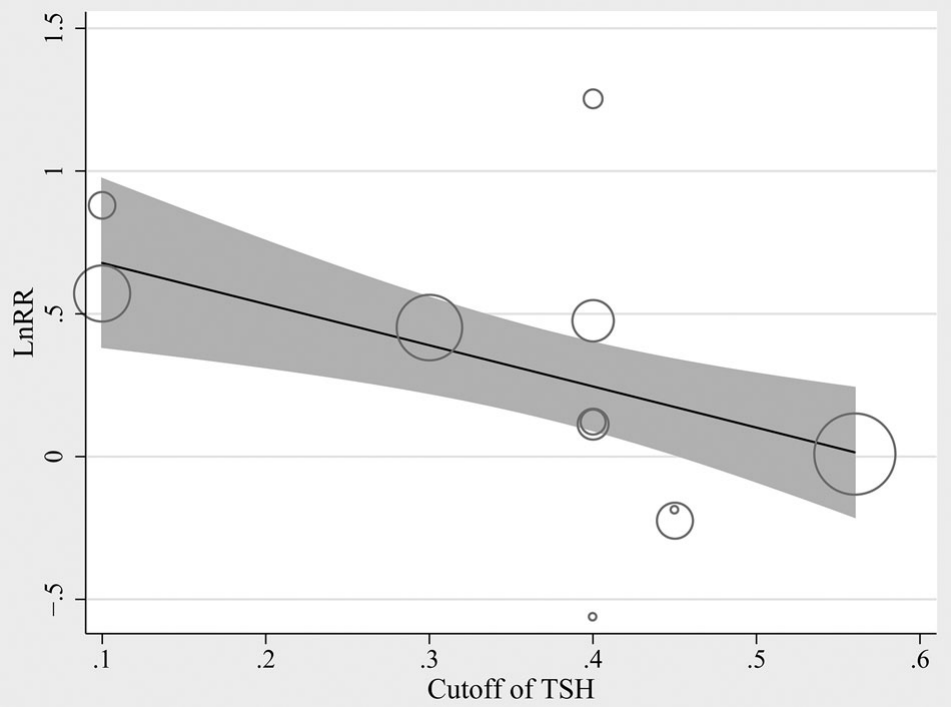
Univariate analysis to evaluate the influence of TSH cutoff used for the diagnosis of subclinical hyperthyroidism on the association between subclinical hyperthyroidism and risk of dementia.

Since two studies reported the data according to the severity of subclinical hyperthyroidism (different cutoffs of TSH) (Aubert et al., 2017; Vadiveloo et al., 2011), these data were included independently into the meta‐analysis. Overall, 11 datasets from nine cohort were included in the meta‐analysis. The pooled results showed that compared to the participants with euthyroidism, those with subclinical hyperthyroidism had a higher incidence of dementia (RR: 1.38, 95% CI: 1.09 to 1.74, *p* = .006; Figure 2A) with moderate heterogeneity (*I* ^2^ = 47%). In addition, pooled results of the two studies (de Jong et al., 2006; Kalmijn et al., 2000) did not show a significant association between subclinical hyperthyroidism and the increased risk of AD (RR: 1.60, 95% CI: 0.36 to 7.06, *p* = .53; *I* ^2^ = 72%; Figure 2B). For the outcome of overall dementia, the sensitivity analysis by excluding one dataset at a time showed consistent results (data not shown). Subgroup analyses of the overall dementia according to study design (*p* for subgroup difference = .34; Figure 3A), age of the participants (*p* for subgroup difference = .88; Figure 3B), methods for diagnosis of dementia (*p* for subgroup difference = .64; Figure 4A), or analytic models (*p* for subgroup difference = .15; Figure 4B) did not significantly change the results. The univariate meta‐regression for the outcome of overall dementia showed that the cutoff of TSH for diagnosis of subclinical hyperthyroidism may negatively affect the association between subclinical hyperthyroidism and dementia (coefficient: −1.44, 95% CI: −2.42 to −0.46, *p* = .009; Figure 5), which completely explained the heterogeneity (residual *I* ^2^ = 0%).

### 3.4 Publication bias evaluation

**FIGURE 6:**
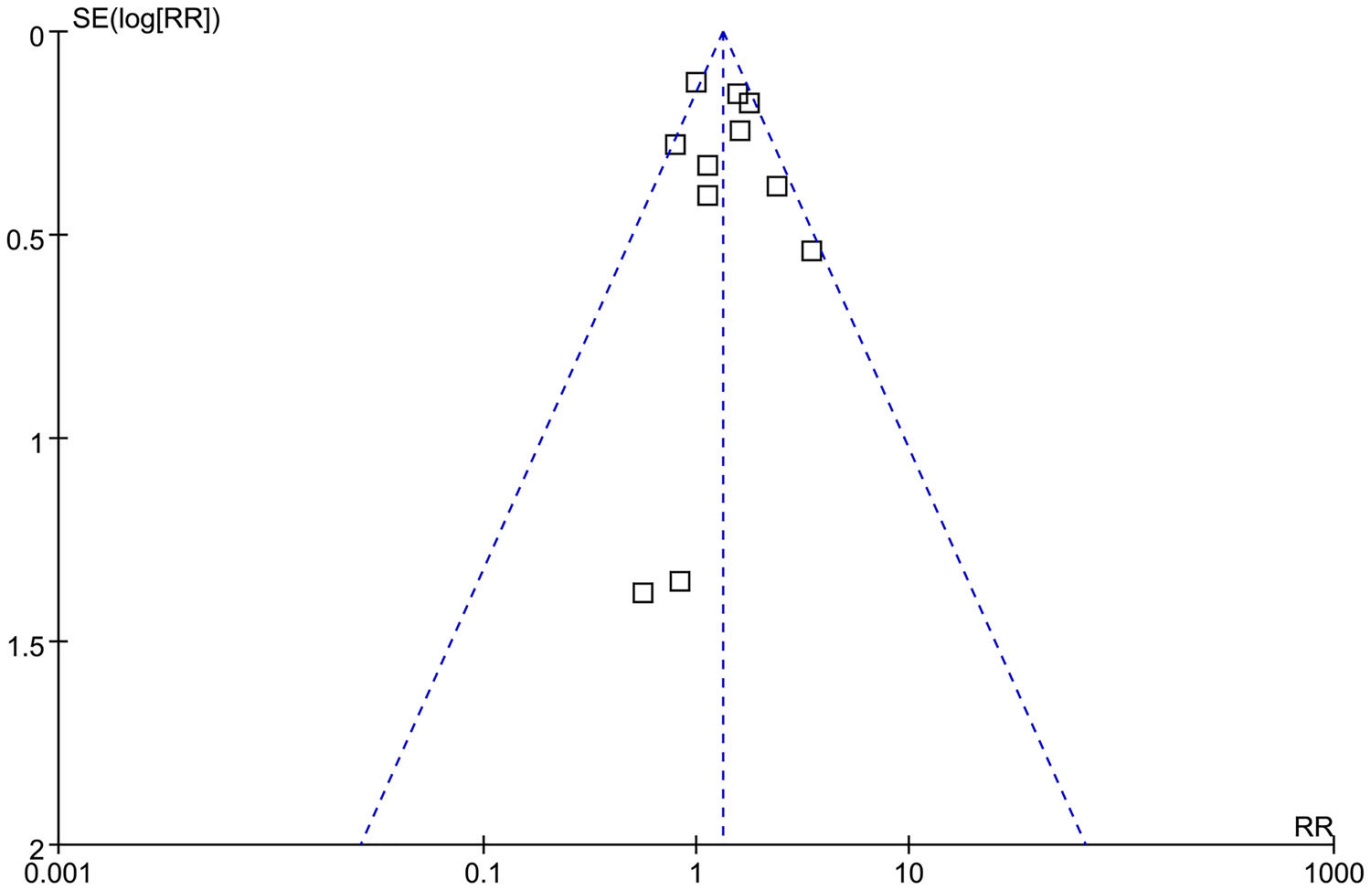
Funnel plots for the publication bias underlying the meta‐analysis of the association between subclinical hyperthyroidism and overall dementia.

The funnel plots for the meta‐analysis investigating the relationship between subclinical hyperthyroidism and the risk of dementia in general adult population is shown in Figure 6 and the symmetrical nature of the funnel plots suggested a low likelihood of publication bias. Results of the Egger's regression test also showed low risk of publication bias underlying the meta‐analysis (*p* = .34).

## 4 DISCUSSION

In this study, we performed a meta‐analysis by incorporating data from the nine cohort studies, to examine the correlation between subclinical hyperthyroidism and the risk of dementia. The results indicated that compared to those with euthyroidism, the participants with subclinical hyperthyroidism were associated with a higher incidence of overall dementia, although limited datasets did not support that subclinical hyperthyroidism was associated with a higher risk of AD. Further sensitivity and subgroup analyses confirmed the robustness and stability of the findings. Moreover, results of univariate meta‐regression suggested that the cutoff of TSH for the diagnosis of subclinical hyperthyroidism was negatively related to the association between subclinical hyperthyroidism and the risk of dementia, which adequately explained the source of heterogeneity. In addition, the findings of the meta‐regression analysis suggested a significant correlation between a lower TSH and a higher RR for the link between subclinical hyperthyroidism and dementia, which implies that the severity of subclinical hyperthyroidism may affect the risk of dementia. As a summary, the combined outcomes of this meta‐analysis provide evidence for a plausible correlation between subclinical hyperthyroidism and the risk of dementia in community‐derived adult population.

As far as we acknowledged, there are limited meta‐analyses, which summarized the relationship between subclinical hyperthyroidism and the risk of dementia. An early meta‐analysis in 2016 included five prospective cohort studies and showed that subclinical hyperthyroidism might be associated with an elevated risk for dementia, while the limited number of the included studies prevented the authors to perform subsequent subgroup and meta‐regression analysis to investigate the influence of study characteristic on the association (Rieben et al., 2016). A recent meta‐analysis in 2023 involved six observational studies and suggested a possible correlation between subclinical hyperthyroidism and increased risk of dementia (Ma et al., 2023). However, this study was based on univariate analysis with limited number of available datasets (Ma et al., 2023). Although the current meta‐analysis showed similar results to the previous ones, it is crucial to recognize the meticulous methodology employed in this meta‐analysis before interpreting the results. Notably, a thorough search of three widely utilized electronic databases was conducted, resulting in the identification of nine contemporary cohort studies with 11 datasets that align with the objectives of this meta‐analysis. Furthermore, only cohort studies were considered, allowing for the examination of a longitudinal relationship between subclinical hyperthyroidism and the incidence of dementia. Additionally, the robustness of the findings was further confirmed through various sensitivity and subgroup analyses, which suggested that the results were neither primarily driven by either of the included datasets not they could be significantly affected by study characteristics such as study design, patient age group, methods for the diagnosis of dementia, or regression models used in the analyses. It is important to notice we showed a significant association between subclinical hyperthyroidism and the risk of dementia in subgroup of multivariate analysis, which therefore suggests that the association may be independent of age, sex, and cardiovascular comorbidities of the participants. This is essential because it has been confirmed that aging (Grande et al., 2020) and cardiovascular risk factors (Nordestgaard et al., 2022) are potential risk factors of dementia. Finally, a univariate meta‐regression analysis suggested that the cutoff of TSH for the diagnosis of subclinical hyperthyroidism may be an important modifier for the association between subclinical hyperthyroidism and the incidence of dementia, which completely explained the source of heterogeneity. The negative correlation found in the meta‐regression analysis also reflected that the association between subclinical hyperthyroidism and the incidence of dementia may be stronger in patients with a lower TSH, suggesting that the severity of subclinical hyperthyroidism may affect the association. These results underscore the significance of thyroid dysfunction in risk stratification of dementia in the general population, and even hyperthyroidism at a subclinical stage could contribute to the risk of dementia.

The mechanisms underlying the association between subclinical hyperthyroidism and the risk of dementia remain largely unknown. One major reason may be that substantial patients with subclinical hyperthyroidism will progress to overt hyperthyroidism, which has been linked to the risk of dementia (Joy Mathew et al., 2020; Khaleghzadeh‐Ahangar et al., 2022). Interestingly, another recent Mendelian randomization study showed that increased levels of genetically predicted TSH within the normal range and in younger individuals are associated with a decreased risk of AD, while a marginal association between genetically predicted full range TSH and AD risk was observed (Marouli et al., 2021). More studies are needed to determine the molecular mechanisms underlying the association between subclinical hyperthyroidism and the risk of dementia, which is fundamental for the prevention of dementia in this specific population.

This study also has some limitations to note. One important issue is that the diagnostic cutoff of TSH for subclinical hyperthyroidism varied among the included studies. However, results of our meta‐regression highly suggested that this is the primary source of heterogeneity. Unfortunately, we are unable to suggest a specific cutoff value for TSH to optimally indicate the risk of dementia at current stage. Large‐scale prospective studies are warranted for further investigation. Furthermore, some of the incorporated studies exhibited a retrospective design, thereby potentially subjecting the outcomes of the meta‐analysis to recall and selection biases. Only the associations between subclinical hyperthyroidism with overall dementia and AD were reported in the included studies; it remains unknown whether subclinical hyperthyroidism is related to a higher incidence of vascular dementia, which requires further investigation. To substantiate the findings of the meta‐analysis, it is imperative to conduct extensive prospective cohort studies on a large scale. Additionally, our study solely encompassed observational studies, thus precluding the establishment of a causal relationship between subclinical hyperthyroidism and the risk of dementia. Consequently, it is crucial to ascertain whether interventions aimed at addressing subclinical hyperthyroidism could reduce the risk of dementia in this population.

## 5 CONCLUSIONS

In conclusion, the findings of the meta‐analysis suggest that compared to those with euthyroidism, the participants with subclinical hyperthyroidism were associated with a higher incidence of overall dementia. Although the mechanisms underlying the association deserve further investigation, these results highlight the importance of the evaluation of thyroid dysfunction in the risk stratification of dementia in the general population, even for those with subclinical hyperthyroidism.

## AUTHOR CONTRIBUTIONS

**Qiao Liu**: Conceptualization; methodology; validation; investigation; writing—original draft; writing—review and editing; formal analysis; data curation. **Chaoyin Lu**: Data curation; methodology; validation; writing—review and editing; visualization; formal analysis. **Mengdie Chen**: Methodology; validation; formal analysis; visualization; writing—review and editing; data curation. **Ping Feng**: Data curation; validation; visualization; conceptualization; investigation; funding acquisition; writing—review and editing; formal analysis; supervision.

## FUNDING

No funding was received for this meta‐analysis.

## CONFLICT OF INTEREST STATEMENT

The authors declare that they have no conflict of interest.

### PEER REVIEW

The peer review history for this article is available at https://publons.com/publon/10.1002/brb3.70037.

## Data Availability

Data sharing is not applicable to this article as no new data were created or analyzed in this study. The data that support the findings are available in the manuscript.
